# The Network of Colonic Host Defense Peptides as an Innate Immune Defense Against Enteropathogenic Bacteria

**DOI:** 10.3389/fimmu.2020.00965

**Published:** 2020-05-20

**Authors:** Graham A. D. Blyth, Liam Connors, Cristina Fodor, Eduardo R. Cobo

**Affiliations:** ^1^Department of Microbiology, Immunology and Infectious Diseases, Cumming School of Medicine, University of Calgary, Calgary, AB, Canada; ^2^Bachelor of Health Sciences, Cumming School of Medicine, University of Calgary, Calgary, AB, Canada; ^3^Department of Production Animal Health, Faculty of Veterinary Medicine, University of Calgary, Calgary, AB, Canada

**Keywords:** colonic epithelium, host defense peptides, cathelicidin, defensin, colitis

## Abstract

Host defense peptides, abundantly secreted by colonic epithelial cells and leukocytes, are proposed to be critical components of an innate immune response in the colon against enteropathogenic bacteria, including *Shigella* spp., *Salmonella* spp., *Clostridium difficile*, and attaching and effacing *Escherichia coli* and *Citrobacter rodentium*. These short cationic peptides are bactericidal against both Gram-positive and -negative enteric pathogens, but may also exert killing effects on intestinal luminal microbiota. Simultaneously, these peptides modulate numerous cellular responses crucial for gut defenses, including leukocyte chemotaxis and migration, wound healing, cytokine production, cell proliferation, and pathogen sensing. This review discusses recent advances in our understanding of expression, mechanisms of action and microbicidal and immunomodulatory functions of major colonic host defense peptides, namely cathelicidins, β-defensins, and members of the Regenerating islet-derived protein III (RegIII) and Resistin-like molecule (RELM) families. In a theoretical framework where these peptides work synergistically, aspects of pathogenesis of infectious colitis reviewed herein uncover roles of host defense peptides aimed to promote epithelial defenses and prevent pathogen colonization, mediated through a combination of direct antimicrobial function and fine-tuning of host immune response and inflammation. This interactive host defense peptide network may decode how the intestinal immune system functions to quickly clear infections, restore homeostasis and avoid damaging inflammation associated with pathogen persistence during infectious colitis. This information is of interest in development of host defense peptides (either alone or in combination with reduced doses of antibiotics) as antimicrobial and immunomodulatory therapeutics for controlling infectious colitis.

## Innate Immunity in Infectious Colitis and the Presence of Host Defense Peptides

Infectious diarrhea causes inflammation of the gastrointestinal tract, clinically manifested by diarrhea, dehydration and, in severe cases, death. Infectious diarrhea is a major cause of morbidity and mortality worldwide, particularly in developing countries (1). Diarrhea is regarded as the 8th leading cause of death, with children (<5 year of age) being responsible for over a quarter of deaths (2). Of the >1 million diarrheal deaths attributed to infectious agents, bacterial pathogens were collectively responsible for ~57% (2). Likewise, of the >2 billion global cases of diarrhea due to foodborne illnesses in 2010, 32% were due to bacteria (3). In addition, bacterial diarrhea is a main cause of illness in travelers seeking medical care after returning from developing nations (4).

The main genera of bacteria that cause infectious colitis are *Escherichia, Salmonella, Campylobacter, Vibrio, Listeria*, and *Shigella*. Increased emergence of antibiotic resistant bacterial strains have limited our ability to treat important enteric pathogens including *Escherichia* and *Salmonella*, and raises the possibility of increased prevalence and mortality due to intestinal bacterial infections (5–7). Indeed, antibiotic resistance is predicted to be a major future public health problem, with antibiotic resistant bacteria expected to cause > 10 million deaths globally by 2050 (8). Development and commercialization of new antibiotics is minimal and there are predictions that without substantial changes, bacterial resistance will continue to increase. Therefore, understanding innate immune mechanisms that aid in pathogen clearance and resolution are critical to understanding pathophysiology of infectious colitis and developing novel antimicrobial and immunomodulatory therapeutics.

The gastrointestinal tract has metabolic functions of digestion and nutrient absorption and also provides a barrier against large numbers of commensal or pathogenic microbes in the lumen. The colon employs multiple innate mechanisms to prevent and clear bacterial infections. For example, MUC2 mucin glycoprotein secreted by goblet cells and host defense peptides (HDPs) secreted by intestinal epithelial cells into the luminal environment compose the mucus layer, an acellular first line of defense. Intestinal epithelial cells, held together by apical junctional complexes, form a second line of defense to prevent penetration of bacteria into the lamina propria. If pathogenic bacteria are able to penetrate mucus and epithelial barriers, underlying leukocytes can protect the host by initiating inflammatory responses to clear invading pathogens.

**Figure 1:**
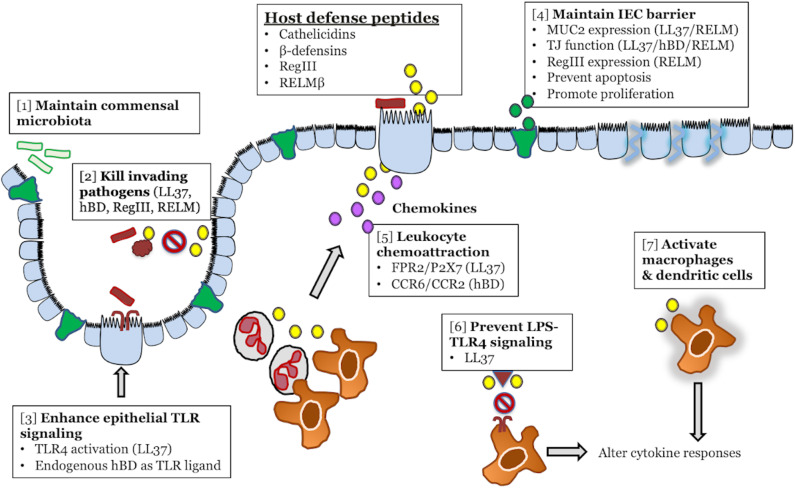
Colonic host defense peptides (HDPs) contribute to intestinal homeostasis and innate immune defense during infectious colitis through multiple overlapping mechanisms. HDPs secreted from intestinal epithelial cells (yellow) exert direct antibacterial effects on both the intestinal microbiota [**1**] and invading bacterial pathogens [**2**]. In terms of immunomodulatory functions, HDPs can enhance the immune signaling capacity of intestinal epithelial cells by forming complexes with LPS/TLR4 (LL-37), or by directly activating TLRs (β-defensins) [**3**]. HDPs might maintain intestinal epithelial cell barrier and prevention of pathogen invasion by increasing MUC2 secretion (green) and tight junction protein expression [**4**]. In the lamina propria, HPDs (yellow) secreted by epithelial cells or infiltrating leukocytes can directly chemoattract leukocytes from the blood (neutrophils, macrophages), through activation of FPR2, P2X7, CCR2, and CCR6, or promote the secretion of chemoattractant (CXCL8/IL-8) by epithelial cells (purple) [**5**]. In tissue resident macrophages, HDPs (yellow) can either promote anti-inflammatory responses by blocking LPS-TLR4 interaction [**6**] or activate macrophages and dendritic cells [**7**] to alter cytokine responses.

Among innate effectors in the colon, HDPs are short cationic peptides abundantly secreted into the lumen by leukocytes and the intestinal epithelium, with key functions in maintenance of gut homeostasis (Figure 1). In the colon, HDPs are mostly represented by cathelicidin, β-defensins, and members of the Regenerating islet-derived protein III (RegIII) and Resistin-like molecule (RELM) families. Known as broad-spectrum antimicrobials, their contribution to innate gut defenses is expected to extend beyond direct lytic effects on bacteria to include immune functions reported *in vitro* (e.g., recruitment of immune cells, wound healing, and cytokine production; Table 1) (9, 42, 43). These immunomodulatory functions have been mostly described for myeloid-derived immune cells (10, 44). A key question is the extent to which immunomodulatory effects of HDPs occur in the gut and their relevance in infectious diseases. There are indications that secreted colonic HDPs are not merely antimicrobial, but also contribute to orchestrated immune responses. Understanding these aspects of HDP function is necessary for identifying novel anti-inflammatory and anti-infective targets as alternatives to conventional antimicrobials. Herein, we review recent advances in our understanding of HDPs in gut innate immune defenses and their role in pathogenesis of infectious colitis.

**Table 1:** Immunomodulatory cellular functions attributed to colonic host defense peptides.

**Host defense peptide**	**Immune function**	**References**
Cathelicidin	• Alter chemokine responses • Inhibit TLR4 activation (leukocytes) • Enhance TLR4 signaling (enterocytes) • Chemoattractant • Increase MUC2 expression • Induce NET formation	(9–23)
β-defensin	• Chemoattractant • Activate dendritic cells and monocytes • Stimulate cytokine release • Increase epithelial barrier function • Induce epithelial cell migration • Induce MUC2 expression	(24–32)
REGIII	• Promote epithelial cell proliferation • Prevent apoptosis	(33, 34)
RELM-β	• Increase epithelial cell proliferation • Chemoattract T-cells • Regulate REGIII expression • Regulate epithelial cell barrier function • Increase mucus secretion • Promote fibrosis	(35–41)

## Cathelicidins

Cathelicidins are small, cationic, amphipathic peptides produced by epithelial cells, macrophages, and polymorphonuclear leukocytes (44, 45). These peptides are synthesized as pro-peptide precursors with a highly conserved N-terminal region (cathelin domain) and a highly variable antimicrobial cathelicidin peptide domain in their C-termini. Cleavage of the C-terminal domain from the holoprotein (e.g., by serine proteases) is required for antimicrobial activity. Humans have a single cathelicidin gene (*cathelicidin antimicrobial peptid*e, *CAMP*), which yields a 37 amino acid peptide (leucine-leucine, LL-37) generated by extracellular cleavage of the C-terminus (46). The murine counterpart is cathelicidin-related-antimicrobial-peptide (CRAMP), encoded by the gene *Camp* (formerly *Cnlp*) (47).

In the colon, cathelicidins are mostly secreted by neutrophils and epithelial cells (42, 48). Differentiated colonic epithelial cells at the peak of crypts constitutively secrete cathelicidins (42, 48, 49), which are normally present in intestinal mucus of healthy individuals (50). Protective roles of cathelicidin in infectious colitis have been demonstrated in mice homozygous for null mutations in *Camp* (*Camp*^−/−^). These mice had exacerbated diarrhea, destructive colitis, and increased pathogen burden after challenge by chemical (43) or infectious (*Clostridium difficile*) (51) agents. Accordingly, *Camp*^−/−^ mice were more susceptible to infection with attaching/effacing bacteria including *C. rodentium* (42) and *E. coli* O157:H7 (11). Consistent with these findings, upregulation of endogenous cathelicidin ameliorated colitis caused by enteropathogenic *E. coli* in rabbits (52).

Signaling pathways that regulate cathelicidin synthesis in the colon respond to both bacterial and endogenous stimuli. Regarding bacterial stimuli, colonic epithelium produced cathelicidins in response to bacterial by-products, such as short chain fatty acids (e.g., butyrate) (48, 53) via MEK-ERK signaling (49, 54). Bacterial DNA also stimulated cathelicidin in colonic lamina propria macrophages through TLR9 (43). This mechanism was observed *in vivo* when intracolonic exposure to *E. coli* genomic DNA upregulated cathelicidin expression in mice via TLR9 signaling (43). Similar to bacterial DNA, double-stranded RNA mimic poly(I:C) induced cathelicidin expression from intestinal epithelial cells via PI3-kinase-PKCζ-Sp1 signaling independent of MAPK pathways (55). MAPK signaling was also required for cathelicidin expression from colonic epithelial cells exposed to a combination of IL-1β and purified MUC2 (56).

Direct antibacterial activity was the first function identified for cathelicidins (57) with most studies focusing on the role of LL-37 against *E. coli*. Whereas, multiple antibacterial mechanisms may occur simultaneously, a principal bactericidal action of cathelicidins is membrane pore formation followed by direct bacterial death. LL-37 recognizes negatively charged lipids, a major component of Gram-negative bacterial membranes (58, 59). Binding of LL-37 to the bacterial surface leads to formation of transmembrane pores that induce bacterial cell lysis (60, 61). This pore formation depends on the alpha-helical amphipathic structure of LL-37, which shape its interactions with negatively-charged and hydrophobic targets on bacterial membranes. Because the structure of LL-37 is highly dependent on environmental factors (e.g., pH and anion concentration), the ability of cathelicidins to kill *E. coli* by transmembrane pore formation may be affected in physiological conditions (61).

Other antimicrobial mechanisms of cathelicidins include binding LPS to cross bacterial outer membranes into the periplasmic space, where LL-37 binds and immobilizes peptidoglycan to impede cell wall biogenesis and growth (62). Aditionally, there is a large influx of LL-37 into the bacterial cell after permeabilization of outer and cytoplasmic membranes that rigidifies the cytoplasm and halts motion of chromosomal DNA and ribosomes, thereby arresting *E. coli* growth (63). The polycationic nature of LL-37 allows it to form a network of electrostatic bonds with polyanionic DNA and ribosomes, preventing proper diffusion of cellular components (63). However, some of these antibacterial effects may be simply bacteriostatic and may not be effective at the population level. In high-density *E. coli* cultures exposed to LL-37, a sub-population of non-growing bacterial cells absorb massive amounts of LL-37 to deplete it from the surrounding environment, enabling a second sub-population to continue growing (64). Unlike LL-37, which interacts directly with microbial cell surfaces [e.g., *E. histolytica* (56)], other cathelicidins seem to internalize within bacterial cells and trigger non-lytic mechanisms. For example, porcine proline rich cathelicidin (PR-39), abundant in myeloid cells in pigs, crosses the cell membrane and likely kills pathogens by blocking bacterial DNA and peptide synthesis (65).

In an attempt to establish infection, intestinal pathogens may actively dampen cathelicidin defenses by multiple strategies. One strategy is to decrease cathelicidin expression in the colon during bacterial colonization. Cathelicidin production was decreased in colonic epithelium and leukocytes of shigellosis patients during early infection, where both live *Shigella* and bacterial plasmids blocked transcription of cathelicidin mRNA (66). Cathelicidin was also transcriptionally suppressed in colonic epithelial cells by exotoxins of *Vibrio cholera* and *E. coli* (cholera toxin and labile toxin, respectively) (67). Thus, cathelicidin silencing is likely a key virulence mechanism used by bacterial pathogens to facilitate intestinal colonization. Another evasion strategy is to repel direct killing by cathelicidins. While cathelicidins displayed *in vitro* killing activity against multiple strains of *E. coli* (11, 68), *Salmonella enterica* serovar Typhimurium resisted killing by cathelicidin through modulation of its outer membrane properties (69). Of note, despite these findings, the real relevance of cathelicidin antimicrobial activity in the gut is still controversial. Cathelicidins showed broad *in vitro* antibacterial activity (either bactericidal or bacteriostatic) against both Gram-positive and -negative bacteria (12, 70–73) (Table 2). However, this antibacterial activity is often abolished under physiological conditions, including presence of high salt concentrations (68, 85), serum (86), plasma alipoprotein-A1 (87), and sugars (88). Antibacterial activity of certain cathelicidins [synthetic cathelicidin (C18G), protegrin, magainin-like peptide] could be further inhibited by bacterial surface modifications, e.g., lipid A acylation by *Salmonella spp*. (89). Therefore, it is still questionable if cathelicidins undertake extensive antimicrobial activities in the colonic lumen. It is possible that microbicidal activities are restricted to certain conditions or niches (e.g., deeper in intestinal crypts, within the inner mucus layer) where cathelicidins can reach high concentrations and/or overcome inhibitory physiological effects.

**Table 2:** Direct *in vitro* antimicrobial functions of colonic host defense peptides.

**Host defense peptide**		**Antibacterial activity**		**References**
		**Gram-negative**	**Gram-positive**	
Cathelicidin		*E. coli* *C. rodentium* *S. enterica* *S. enteritidis* *K. pneumoniae*	*L. monocytogenes* *S. aureus* *E. faecalis*	(12, 42, 70–73)
β-defensin	-1	*E. coli* *S. enteritidis*	*Bifidobacterium* spp. *Lactobacillus* spp. *B. subtilis* *S. aureus*	(29, 74)
	-2	*E. coli* *P. aeruginosa* *H. pylori*	*S. aureus* *S. pyogenes*	(75–78)
	-3	*S. enterica* *E. coli* *P. aeruginosa*	*L. monocytogenes* *S. aureus* *E. faecalis*	(78)
	-4	*E. coli* *P. aeruginosa*	*S. aureus*	(79)
RELM-β		*P. aeruginosa* *C. rodentium*	*L. monocytogenes* *E. faecalis*	(80)
REGIII	-β	*E. coli* *Bacteroides* spp.		(81, 82)
	-γ		*L. monocytogenes* *L. innocua* *E. faecalis*	(83, 84)

On the other hand, there is growing evidence that immunomodulation is a critical function of cathelicidins in gut homeostasis. Such immunomodulation can be achieved by signaling through both colonic epithelial cells and immune cells, often at concentrations lower than is required for antimicrobial activity (Figure 1, Table 1). A first role of cathelicidins in gut innate immuninty could be enhancement of Toll-like receptor (TLR) sensing and prevention of pathogen invasion into colonic epithelial cells. For instance, human adenocarcinoma colonic epithelial (HT-29) cells exposed to a combination of synthetic LL-37 and LPS had increased TLR4 gene and protein expression (13). Such TLR4 activation is expected to increase production of pro-inflammatory cytokines, since LL-37 was required for CXCL8 and IL-1β production from colonocytes exposed to bacterial stimuli (13, 14). Moreover, the combination of cathelicidin and LPS prevented invasion of *Salmonella enterica* serovar Typhimurium into HT-29 cells (14). Although the synergistic effect between cathelicidins and LPS has not be tested *in vivo*, HT-29 cells served to examine colonic epithelial cell responses, as they constitutively express TLR4 and secrete CXCL8 in response to LPS as do primary intestinal epithelial cells (90). In support of this presumptive role in the colonic mucosa, LL-37 primed inflammatory responses in airway epithelial cells during *Pseudomonas aeruginosa* infection, promoting IL-1β and IL-18 secretion in an NLRP3 and caspase-1 dependent fashion (91).

Other immunomodulatory roles of cathelicidins in the gut include regulation of the intestinal epithelial barrier. This is important because the epithelial barrier plays a critical role in colonic histopathological changes and diarrhea that characterize infectious colitis. The epithelial paracellular barrier is largely maintained by tight junctions (TJs), and is critical for water absorption and restricting invasion of enteric luminal bacteria. TJs are disrupted in the colon exposed to enteric pathogens (92), but cathelicidins induced TJ gene expression in mammalian enterocytes and porcine intestines (9, 93). In addition, LL-37 prevented disruption of the TJ protein ZO-1 during *S. enterica* serovar Typhimurium infection in colonic epithelial (T84) cells (14). Effects of cathelicidins are not restricted to gut epithelium, as LL-37 induces upregulation of tight junction proteins and increases epithelial barrier function in keratinocytes (94). Although significance of cathelicidins in maintaining the gut barrier is incompletely understood, these functions might contribute to the increased pathogen burden and histopathological damage in *Camp*^−/−^ mice infected with *C. rodentium* (42).

The colonic epithelial barrier is also maintained by the mucus layer, mainly composed of MUC2 mucin derived from intestinal goblet cells (56). MUC2 mucin limited *in vitro* colonization by pathogenic *E. coli* (95) and mechanically expelled pathogens from the gut to prevent *C. rodentium* propagation (96). The colonic mucus barrier is comprised of a firmly attached inner layer devoid of bacteria and a more loosely attached outer layer which contains large numbers of commensal bacteria (97). *Camp*^−/−^ mice had a thinner colonic mucus layer and were more easily penetrated by *E. coli* O157:H7 (11), demonstrating the importance of cathelicidin for forming an effective mucus barrier. Moreover, stimulation of HT-29 cells with LL-37 induced gene expression of mucins *MUC1* and *MUC2* via the P2X purinoceptor 7 (P2X7) and MAP kinase pathway (9, 15). This function has been demonstrated in other epithelia, where stimulation of airway epithelial (NCI-H292) cells with LL-37 resulted in MUC5AC production through EGFR activation (98). Thus, it has been postulated that cathelicidins and mucin coexist as first line defenders in the intestinal lumen. Moreover, the more compact inner mucus layer could retain cationic peptides due to its overall negative charge and provides a gradient of antimicrobial HDPs that separates commensal microbiota from the epithelium (50). Indeed, cathelicidins are implicated in maintaining the colonic microbiota. *Camp*^−/−^ mice display a different colonic microbiota in comparison to *Camp*^+/+^ mice, mostly associated with increased populations of *Odoribacter lanues, Desulfovibrio piger*, and *Desulfomicrobium orale* in *Camp*^−/−^ mice (99).

It is known cathelicidins can act as direct chemoattractants to promote leukocyte influx to the site of infection; a role that could be critical in infectious colitis (100, 101). In leukocytes, cathelicidins signal through a range of receptors, including P2X7 and Formyl Peptide Receptor 2 (FPR2) that recognize extracellular ATP and N-formyl peptides, respectively. Accordingly, LL-37 inhibited neutrophil apoptosis by signaling through P2X7 and PI3K pathway (102), and chemoattracted FPR2-expressing peripheral blood monocytes, neutrophils and CD4^+^ T cells (16). The inhibition of apoptosis in neutrophils was abrogated by blocking P2X7 and the PI3K pathway (102), while the chemoattractant function of cathelicidin was inhibited by both a specific FPR2 inhibitor and the G-protein coupled receptor inhibitor pertussis toxin (16). Moreover, LL-37 directly activated CD11b/CD18 to increase monocyte migration (103) and phagocytosis of LL-37 coated bacteria (104), indicating a role of cathelicidin in leukocyte phagocytosis and migration through CD11b/CD18. Indirect activation of CD11b/CD18 by LL-37 and CRAMP on monocytes can also occur through activation of FPR2 (105). Cathelicidins can promote additional antimicrobial functions in neutrophils, such as induction of neutrophil extracellular trap (NET) formation. Stimulation of human neutrophils with both LL-37 and CRAMP resulted in an increase in NET formation (17, 18). Further research is needed to define the importance of these chemoattractant and pro-phagoctytic effects of cathelicidins in gut physiology and defenses in infectious colitis.

One intriguing aspect of cathelicidins is their pleiotropic nature, exerting either pro-inflammatory effects or attenuating inflammation depending on the environment. Cathelicidins inhibit LPS-induced pro-inflammatory responses in leukocytes. LL-37 inhibited the LPS-induced secretion of TNF-α from phagocytic THP-1 cells (10) by blocking binding of LPS to CD14 (19). This LPS neutralization was also important for preventing LPS-induced apoptosis in endothelial cells (106) and LPS/ATP-induced macrophage pyroptosis (20). Likewise, LL-37 lowered *TNF-*α*, Cxcl-1*, and *IL-1*β expression in both cultured murine macrophages and mammary epithelial cells exposed to the pathogenic algae *Prototheca bovis* (107). On the other hand, cathelicidins seem to promote inflammatory responses in intestinal epithelium (13, 14). Cathelicidin exhibited pro-inflammatory functions in intestinal epithelial cells exposed to LPS or *S. enterica*, including increased TLR4 expression, increased CXCL8 expression, and increased IL-1β (13, 14). It is likely that pro- or anti-inflammatory immunomodulatory function of cathelicidin is cell-type specific, and depends on the receptors expressed by either leukocytes or epithelial cells, infection status, the class of infecting pathogen, and the surrounding cytokine milieu.

The inter-species activity of cathelicidins is of interest for understanding the ontogeny of these ancenstral defenses and the development of therapeutics. Although all cathelicidins share a highly conserved N-terminal cathelin domain, the C-terminal antimicrobial domain is highly variable, both in sequence identity and secondary structure (45). For example, LL-37 and CRAMP share <70% sequence identity, however, they are still considered homologous. This is because both LL-37 and CRAMP share similar structure (α-helical and net charge of +6), antimicrobial capabilities (12, 108), and show interspecies functional capacity (109, 110). Both peptides comparably regulated chemokine expression and TLR 4 activity in myeloid cells (12, 21, 22) and neutrophil recruitment via FPR2 (16, 23). Moreover, mice infected with H1N1 influenza A virus had enhanced survival and reduced viral titer upon treatment with nebulized LL-37 (109). Likewise, intranasal administration of LL-37 increased inflammatory responses in sinonasal tissue of mice (110), while CRAMP was chemoattractant for human monocytes, neutrophils, and macrophages (23). However, an analysis of the interspecies functionality of 12 cathelicidins from 6 different species showed varying immunomodulatory activity results. While cathelicidins from all species demonstrated antimicrobial and LPS neutralizing function, there was large variability in the peptides' abilities to induce cytokine secretion from RAW264.7 macrophages (12). Thus, although they share common aspects, each cathelicidin should be studied as a unique peptide with specific activities in each host.

Given the antimicrobial and immunomodulatory characteristics of endogenous cathelicidins, the use of exogenous cathelicidin peptides (or their derivatives) as therapeutics for infectious colitis is appealing. Systemically administered exogenous cathelicidins were shown to attenuate colitis and reduce *Salmonella* burden in mice (111), while intracolonic CRAMP administration attenuated murine *C. difficle* colitis (51). Furthermore, intraperitoneal injection of porcine cathelicidin PR-39 in EHEC-infected mice improved survival, attenuated inflammatory cell infiltration and pro-inflammatory cytokine production (IL-1β, TNF-α, and IL-6) in the colon, and restored jejunal tight junction formation (112). Treatment of EHEC-infected mice with a cathelicidin derived from the snake *Bungarus fascia* (cathelicidin-WA) was similarly effective as the antibiotic enrofloxacin for increasing survival, reducing histopathological colonic damage and attenuating inflammatory colonic IL-6 production (113). Moreover, cathelicidin-WA was more effective than enrofloxacin for restoring jejunal mucus thickness and goblet cell number in EHEC-infected mice (93). A CRAMP-vancomycin conjugate demonstrated increased antibacterial activity against Gram-negative bacteria when compared to vancomycin or CRAMP alone, or to a 1:1 mixture of vancomycin and CRAMP (114). Synthetic HDPs derived from bovine cathelicidin peptide sequences with direct bactericidal and immunomodulatory functions (named immune defense regulator peptides, IDRs) have been developed for the treatment of diverse bacterial infections (115). IDR-HH2,−1002, and−1018 stimulated neutrophil functions including chemokine secretion, expression of adhesion molecules and release of antimicrobial HDPs, resulting in increased neutrophil killing of *E. coli* (116). IDR-1002 showed anti-inflammatory functions in a murine ear sterile inflammation model, decreasing IL-6, MCP-1, and KC production in PMA inflamed ears (117). Treatment of *P. aeruginosa* infected mice with IDR-1002 showed decreased bacterial burden and associated inflammation, including decreased IL-6 and MCP-1 in bronchoalveolar lavage fluid (118). Additionally, RAW 264.7 macrophages pre-treated with IDR-1002 and then stimulated with LPS showed reduced TNF-α and COX-2 expression (119). IDRs could also be combined with conventional antibiotics; IDR-1018 demonstrated anti-biofilm activity against *P. aeruginosa* and synergistic capabilities with antibiotics to kill biofilms of *P. aeruginosa, E. coli, Acinetobacter baumannii*, and *S. enteria*, among others (24). Thus, cathelicidins show promise as a potential future therapeutic against infectious colitis to reduce or replace antibiotics.

## β-defensins

β-defensins are small cationic HDPs characterized by their cysteine-rich nature and disulphide bridges. There are at least 48 human β-defensin (hBD) genes (120), including hBD-1, -2, -3, and -4 that are highly expressed in the colon (25, 121). In mice, murine β-defensin (mBD)-1, -4, and -14 have been proposed as orthologous to hBD-1, -2, and -3, respectively (120). In terms of gut regulation, hBD-1 is constitutively expressed in colonic epithelium but does not appear to be upregulated by inflammatory signals (26), whereas hBD-2, -3, and -4 are minimally expressed in healthy colonic epithelium but are induced during inflammation (27–29).

Specific pro-inflammatory cytokines regulate colonic β-defensins. For example, IL-1α/β, and TNF-α enhanced expression of hBD-2 in intestinal epithelial cells without affecting hBD-1 expression (26). Such β-defensin induction occurred mostly through NF-κB (26). Likewise, activation of TLR2 and TLR4 directly activated hBD-2 expression in colonic epithelial cells through NF-κB and AP-1 (30), as well as activation of Nucleotide-binding Oligomerization Domain-like Receptor 2 (NOD2) (31). NF-κB-independent mechanisms have also been involved in β-defensin synthesis. Corticosteroids increased β-defensin expression independent of NF-κB in intestinal epithelial (Caco-2) cells (32). Additionally, hBD-3 was upregulated independently of NF-κB in human colonic epithelial cells exposed to extracts from medicinal plants (andrographolide, oridonin, and isoliquiritigen) (122). This upregulation of β-defensin increased bactericidal activity against *Listeria monocytogenes* and, bacteriostatic activity against *S. enterica* in supernatants from human colonic epithelial cells (122). The colonic mucus layer is also important in hBD-2 regulation. The major colonic secretory mucin, MUC2, upregulated hBD-2 in HT-29 cells in response to IL-1β (75). Moreover, mice genetically deficient in Muc2 (*Muc2*^−/−^) had decreased expression of mBD-4 and mBD-14 (75). Interestingly, fully glycosylated mature MUC2 reduced antibacterial activity of hBD-2 against pathogenic (EPEC) and commensal *E. coli*, indicating mucin may protect enteric bacteria from killing by β-defensins (75). These results are particularly important for ulcerative colitis patients, who commonly have diminished or disrupted intestinal mucus layers, suffer from dysbiosis, and are more prone to *Clostridium difficile* infection (123, 124).

Direct antibacterial functions of β-defensins are attributed to a disruption of microbial membranes by pore formation, causing release of intracellular contents and death (125). β-defensin homologs have a broad range of antibacterial activity (Table 2). For instance, hBD-2 has bactericidal activity against Gram-negative bacteria (i.e., *E. coli, P. aeruginosa, Helicobacter pylori*) and fungicidal activity against yeast (*Candida albicans*), but is merely bacteriostatic against the Gram-positive bacterium *Staphylococcus aureus* (76, 77). Conversely, hBD-3 is directly bactericidal against *S. aureus* and vancomycin-resistant *Enterococcus faecium* (VRE) (78). hBD-4 has bactericidal activity against both Gram-negative *E. coli* and *P. aeruginosa* in addition to Gram-positive *S. aureus* (79). Interestingly, reduction of disulphide-bridges in hBD-1 increases its bactericidal activity against *C. albicans* and Gram-positive commensal bacteria (29). Noteworthy, hBD-1 may have additional antibacterial functions beyond direct bacterial lysis; hBD-1 forms an entrapping net that abolished bacterial translocation across polycarbonate membranes and would prevent bacterial invasion (74).

β-defensins have chemoattractant function, although these roles in colitis are poorly defined. Both human (hBD-1, -2, and -3) and murine (mBD-4 and -14) β-defensins induce chemotaxis in leukocytes in a CCR6 dependent fashion (126–129). Moreover, hBD-2 and -3, and the orthologous mBD-4 and -14 induce migration of monocytes, macrophages and neutrophils through interactions with CCR2 (25). β-defensin immunomodulatory function also includes maturation and activation of leukocytes. mBD-2 activates immature dendritic cells, functioning as a TLR4 ligand and upregulating co-stimulatory molecules toward to a TH1 polarized response (130). In addition, hBD-3 activates monocytes in a TLR1/2 dependent fashion (131), whereas hBD-2 and -3 increase pro-inflammatory cytokine release from TLR-stimulated macrophages by ATP release and P2X7 activation (132). Interestingly, hBD-1, -2, and -3 all stimulate cytokine release from human peripheral blood mononuclear cells, with each β-defensin stimulating a unique array of cytokines (133). The presence of hBD-1 in human plasma (134), and expression of hBD-1 and -2 by human monocytes indicates systemic functions of β-defensins (135).

At the gut mucosa, β-defensins regulate epithelial cell responses including proliferation and migration, which are critical for resolution of injury, infection, and inflammation. hBD-2 signals through CCR6 on colonic epithelial (Caco-2 and T84) cells to induce actin cytoskeleton rearrangements and promote cell migration (136). Likewise, hBD-2 increased cell migration, induced *MUC2* transcription and protected against apoptosis in Caco-2 and HT-29 cells (137). Studies in other epithelia infer hBDs may additionally have a role in regulation of intestinal epithelial permeability. hBD-3 improved keratinocyte barrier function through upregulation of tight junction proteins (138). Overall, protective mechanisms of β-defensins during intestinal infection include direct bacterial killing and regulatory functions on immune and intestinal epithelial cells.

## Regenerating Islet-Derived Protein (Reg) III

The Regenerating islet-derived protein III (RegIII) proteins are C-type lectins, ~16–17 kD (139), with the capacity of binding bacterial carbohydrate motifs, independent of calcium, to mediate pore formation in bacterial membranes (140). The *Reg* gene family was originally identified from the *Reg* gene expressed in rat pancreatic regenerating islets (141). A large *Reg* gene family was later characterized and separated into 4 subgroups (I-IV), based on DNA and protein sequence similarity (142, 143). In humans, the *Reg* family consists of 5 genes [*REGI*α, *REGI*β, Hepatocarcinomal-Intestine-Pancreas/Pancreatitis-Associated Protein (*HIP/PAP), REGIII*γ, and *REGIV*], whereas 7 *Reg* genes are present in mice (*RegI, RegII, RegIII*α, *RegIII*β, *RegIII*γ, *RegIII*δ, and *RegIV*) (144). In intestines, *RegIII* genes are the most prevalent *Reg*, with higher expression in the small intestine (83, 145, 146). Mice express 4 *RegIII* family members (*RegIII*α*, -*β, -γ, and -δ*)*, whereas humans express 2 *RegIII* genes (*HIP/PAP* (*REGIII*α*)* and *REGIII*γ*)* with certain homologies (147, 148). Human HIP/PAP and murine RegIIIγ are orthologous and share 67% homology, whereas human REGIIIγ shares 68% homology with murine RegIIIβ (149).

*RegIII* gene expression is increased during intestinal inflammation, as observed in IBD patients and mice afflicted with DSS-induced colitis (*HIP/PAP* and *RegIII*γ) (145). *RegIII* expression in non-hematopoietic cells is mainly induced by activation of pattern recognition receptors and MyD88 signaling. Such innate upregulation of *RegIII*γ in intestinal epithelium conferred protection against *L. monocytogenes* infection (150). Likewise, oral LPS upregulated *RegIII*γ through TLR4 in antibiotic-treated mice, providing increased resistance to vancomycin-resistant *Enterococcus* (VRE) (151). Specialized intracellular nucleotide-binding oligomerization domain-like receptors (NOD-like receptors) also regulate *RegIII*. Mice deficient in Nod (Nod1^−/−^/Nod2^−/−^) had decreased expression of colonic *RegIII*γ, associated with increased susceptibility to DSS-induced colitis (152).

Expression of *RegIII*γ and *RegIII*β in colonic epithelial cells can also be regulated by IL-22 via STAT3 (153, 154). Indeed, mice genetically deficient in STAT3 have delayed wound healing during DSS-colitis, associated with decreased *RegIII*γ and *RegIII*β expression in intestinal epithelial cells (155). Likewise, enteric infections could regulate *RegIII* via IL-22. *RegIII*γ is upregulated during *C. rodentium* infection in response to IL-22 (154), whereas the TLR5 ligand flagellin increased expression of *RegIII*γ in intestinal epithelial cells through IL-22 induction and protected against VRE infections (156). A main source of IL-22 is Th17 cells that regulate *RegIII*γ in human and murine colonic epithelial cells (154, 157). Therefore, upregulation of intestinal *RegIII* can be mediated through cytokines, such as IL-22, or potentially through recognition of pathogen associated molecular patterns, e.g., LPS or flagellin. Collectively, these functions could work to drive pathogen clearance and restore homeostasis.

Antimicrobial functions of RegIII in colonic defense were recently demonstrated (Table 2). *In vitro*, HIP/PAP kills Gram-positive bacteria (*L. monocytogenes* and *E. faecalis*) through formation of an oligomeric pore in the bacterial membrane, although HIP/PAP failed to kill Gram-negative bacteria (83, 84). Unlike other C-type lectins, HIP/PAP binds to bacterial peptidoglycan in a calcium-independent fashion, and bacterial killing requires a conserved EPN motif (140). Accordingly, *RegIII*γ^−/−^ mice have increased abundance of Gram-positive mucosa-associated commensal bacteria (158). Interestingly, whereas RegIIIγ disrupts Gram-positive bacterial membranes, RegIIIβ preferentially kills Gram-negative bacteria (81, 82, 159). The ability of RegIIIβ to specifically target Gram-negative bacteria is due to its ability to bind to carbohydrate motifs of lipid A, a main component of LPS (159). The antibacterial function of RegIIIγ and RegIIIβ to preferentially target Gram-positive and Gram-negative bacteria, respectively, offers an interesting mechanism to for these two related HDPs to jointly protect against different types of bacterial pathogens. The antibacterial activity of RegIIIγ and HIP/PAP is activated through proteolytic removal of an N-terminal pro-segment by trypsin; the protein is inactive until it is secreted into the intestinal lumen and proteolytically processed to generate an active peptide (160). This fine control of RegIIIγ activity is similar to other regulatory mechanisms described for small intestinal α-defensins that require activation by the metalloproteinase matrilysin or trypsin (161, 162).

Roles of RegIII proteins in gut homeostasis may extend beyond direct bactericidal functions (Table 1). In the skin of psoriasis patients, HIP/PAP is highly expressed via IL-17 and promotes keratinocyte proliferation through engagement of exostosin-like 3 (EXTL3) and activation of the PI3K-AKT signaling pathway (33). Some of these immunomodulatory roles for RegIII proteins could apply to the gut, including preventing apoptosis. Treatment of HT-29 cells with recombinant HIP/PAP protected against apoptosis (34). Moreover, IL-22 protected mice from intestinal stem cell apoptosis during graft-vs.-host disease (GVDH) through upregulation of *RegIII*γ (34).

Intestinal microbiota is an important regulator of RegIII. *RegIII*γ genes were upregulated in the colon of germ-free mice upon bacterial colonization (83), and specific commensal bacteria induce *RegIII*γ genes in the colon. Monocolonization of germ-free mice with Gram-positive *Bifidobacterium breve*, but not Gram-negative *E. coli* JM83, increased *RegIII*γ production (149). Similarly, specific pathogen free (SPF) Nod1^−/−^/Nod2^−/−^ mice showed decreased *RegIII*γ expression, which was restored with altered Schaedler flora (ASF) (in gnotobiotic Nod1^−/−^/Nod2^−/−^ mice) or *B. breve* (in SPF Nod1^−/−^/Nod2^−/−^ mice) (152). In addition, monocolonization of germ-free SCID mice with segmented filamentous bacteria (SFB), strong inducers of Th17 cells and mucosal IL-22, increased *RegIII*γ expression in intestinal epithelial cells (163). Likewise, monocolonization of germ-free mice with Gram-negative *Bacteroides thetaiotaomicron* resulted in a modest upregulation of *RegIII*γ (2.5-fold), whereas monocolonization with Gram-positive *Listeria innocua* had no effect on *RegIII*γ expression (83). Interestingly, monocolonization of Rag-1^−/−^ germ-free mice with *B. thetaiotaomicron* and *L. innocua* resulted in a large (40 to 50-fold) increase in *RegIII*γ expression, respectively (83). This phenomenon is hypothesized to be due to the absence of luminal IgA, which normally sequesters commensal bacteria away from intestinal epithelial cells, suggesting contact between bacteria and intestinal epithelial cells is a major driver of *RegIII*γ expression (83).

It has been proposed that RegIIIγ antimicrobial activity is critical to separate commensal bacteria from underlying intestinal epithelial cells (158), or modulate the intestinal microbiota population when stimulated by intestinal infection or inflammation. In fact, RegIII-mediated alteration of the intestinal microbiota can have implications on outcomes of infectious colitis. *RegIII*β expression resulted in prolonged and worsened disease in a streptomycin murine model of *Salmonella*-induced colitis, corresponding with decreased presence and re-establishment of commensal *Bacteroides* spp. (81). Similarly, the regulation of RegIII by the microbiota has implication on infections, as depletion of the intestinal microbiota by antibiotic treatment decreased intestinal *RegIII*γ expression and increased VRE burden (151). Production of RegIIIγ varies across the gut, with lower *RegIII*γ expression in colon compared to small intestine (146); this difference may account for differential microbiome-host interactions. Whereas, *RegIII*γ^−/−^ mice did not display increased commensal bacteria/epithelial cell contact in the colon, they had more intimate bacterial contact in the terminal ileum associated with increased production of inflammatory IL-22 and myeloperoxidase (164). The antibacterial function of RegIIIγ is thought to be of particular importance due to its presence within the mucus layer, preventing penetration of both commensal and pathogenic bacteria. The mucus layer contains RegIIIγ (164) and its larger size compared with other HDPs (e.g., defensins or cathelicidins) could favor closer interactions with mucus glycoproteins thereby preventing RegIIIγ diffusion from the mucus layer into the lumen (158). Thus, RegIII may contribute to gut homeostasis via direct antibacterial functions against intestinal bacterial pathogens, microbiome regulation and immunomodulation (Figure 1).

## Resistin-Like Molecules (RELM)

Resistin-like molecules (RELMs) are a family of secreted proteins characterized by a conserved cysteine-rich C-terminus (165). Previously named Found in Inflammatory Zone (FIZZ) and Hypoxia-Induced Mitogenic Factor (HIMF) proteins (166), the RELM protein family has been studied in a wide range of diseases, including asthma, diabetes, and bacterial and parasitic infections. RELM proteins range in size from 105 to 114 amino acids, with 3 conserved domains: a signal sequence, a variable middle domain, and a C-terminal domain (165). The latter is particularly rich in cysteine residues, invariantly spaced following the sequence C-X_11_-C-X_8_-C-X-C-X_3_-C-X_10_-C-X-C-X-C-X_9_-CC-X_3−6_ (165). Mice have 4 Relm proteins (RELMα, -β, -γ, and resistin) encoded by *Retlna, Retlnb, Retlng*, and *Retn* genes, respectively, whereas humans possess only RELMβ and Resistin, encoded by *RETLNB* and *RETN* genes (165, 167). Murine RELMα is mostly restricted to airway epithelial cells (168) and immune cells (i.e., macrophages and dendritic cells) (169, 170), while murine RELMγ is expressed in bone marrow, spleen, and lungs (171). Human Resistin is expressed in immune cells (172), whereas murine Resistin is restricted to adipocytes (173). Resistin and RELMβ form large hexamers consisting of 2 trimers, linked by disulphide bonds present in their N-terminal coiled-coiled domains (174), although RELMβ may also exist as secreted dimers (175).

RELMβ is the most abundantly expressed RELM in the large intestine of both humans and mice (176), mostly in cecum and distal colon (176). The peptide is produced as a 111-amino acid protein with an N-terminal 11-amino acid signal sequence, and is predominantly restricted to secretory granules of mucus-secreting goblet cells (176). Unsurprisingly, RELMβ expression is regulated by factors that influence goblet cell functions, e.g., IL-18 and IL-22 (177). Expression of RELMβ is dependent on the microbiota, being transcriptionally induced in germ-free mice upon colonization with a conventional microbiota (176). Moreover, oral antibiotic treatment of mice decreased *Firmicutes*, with persistence of *Bacteroidetes* and *Proteobateria*, and decreased RELMβ production, along with decreased IFNγ and IL-17 production from CD4+ T-cells (178).

RELMβ is particularly reactive to helminth intestinal infections, including *Trichinella muris, Trichuris spiralis*, and *Nippostrongylus brasiliensis* as a part of a TH2-driven immune response, mainly mediated through IL-4 and IL-13 (179). Accordingly, RELMβ-deficient mice were more susceptible to *Heligmosomoides polygyrus* and *Nippostrongylus brasiliensis* infections (180). Detection of RELMβ in feces of mice with gastrointestinal nematode infections has also been demonstrated as a non-invasive tool to assess intestinal changes in response to intestinal infections (181). Mechanistically, RELMβ is necessary for IL-4-mediated intestinal worm expulsion, impairing the ability of worms to feed on host tissues and generate ATP (180). In addition, RELMβ can directly bind chemosensory components of parasitic nematodes to block their sensory functions (179).

Colonic RELMβ expression is increased during bacterial infection (e.g., *C. rodentium*), with secreted RELMβ present in feces (35). Indeed, RELMβ deficient mice are more susceptible to *C. rodentium* infection, with decreased survival and increased bacterial colonization deep within colonic crypts (35). Direct microbial killing may be a key role of RELMβ in this gut defense. RELMβ causes pore formation and bacterial death in both Gram-positive and Gram-negative bacteria, including *L. monocytogenes, E. faecails, C. rodentium*, and *P. aeruginosa* (80) (Table 2). Although it is broad-spectrum bactericidal, RELMβ preferentially kills Gram-negative bacteria, with antibacterial functions observed for both monomeric and dimeric forms of the protein (80). Additionally, colonic RELMβ may protect against *C. rodentium* indirectly, by promoting intestinal epithelial cell (IEC) proliferation and chemoattracting T-cells. RELMβ^−/−^ mice infected with *C. rodentium* had increased mortality, with reduced CD4+ T-cell infiltration, reduced IL-22 production, and impaired IEC proliferation in colons (35). Interestingly, RELMβ^−/−^ mice displayed decreased expression of RegIII C-type lectins (36, 37). Thus, decreased CD4+ T-cell infiltration and IL-22 production in the colons of RELMβ^−/−^ mice may be major drivers of decreased *RegIII*γ gene expression, as IL-22 is a strong inducer of RegIIIγ production (153, 154).

RELMβ expression is induced in mice during DSS colitis, with increased expression requiring IL-13, as IL-13^−/−^ mice were unable to induce RELMβ (36). Some pro-inflammatory roles for RELMβ are postulated based on decreased colitis in RELMβ^−/−^ mice exposed to DSS (e.g., decreased weight loss, colonic shortening, and mortality) (36). However, in the same study, RELMβ^−/−^ mice were more susceptible to TNBS-induced colitis (36). These apparent conflicting results are likely due to the underlying inflammatory mechanisms behind these 2 models of colitis. While DSS colitis causes inflammation driven by direct erosion of the epithelial barrier, TNBS-induced colitis is mediated by potent TH1 immune responses (182). In addition, RELMβ^−/−^ mice responses to DSS and TNBS-induced colitis could be regulated by RegIIIβ expression, which showed to reduce TNF-α induced immune responses in monocytes and epithelial cells (36, 183). Thus, RELMβ could undertake either pro- or anti-inflammatory roles depending on the inflammatory stimuli or surrounding immune activation.

RELMs may enhance gut mucosal barrier defenses against pathogenic bacteria by promoting colonic mucin. RELMβ upregulated MUC2 and increased secretion of MUC5AC in mucin-producing colonic epithelial (HT29-Cl.16E) cells, signaling through calcium and PKC (38). Moreover, mice pre-treated with synthetic RELMβ experienced increased mucus production and attenuated TNBS colitis (38). However, RELMβ could still exert pro-inflammatory roles beyond a mucin secretagogue effect. Mice genetically deficient in Muc2 (*Muc2*^−/−^) developed spontaneous colitis, an effect that was diminished in mice double knock out for Muc2 and RELMβ (*Muc2*^−/−^/RELMβ^−/−^) (37). Interestingly, RELMβ expression in *Muc2*^−/−^ mice induced expression of both RegIIIγ and RegIIIβ, which corresponded to a decrease in *Lactobacilli* spp. (37). It is likely that spontaneous colitis in *Muc2*^−/−^ mice responds to replenishment of *Lactobacilli* spp. and a pro inflammatory role of RELMβ affecting healthy intestinal microbiota. Furthermore, the presence of RELMβ in *Muc2*^−/−^ mice could contribute to increased RegIII expression, resulting in microbial dysbiosis and more severe colitis in comparison to *Muc2*^−/−^/RELMβ^−/−^ mice (37). Effects of RELMβ on gut permeability could also impact intestinal homeostasis. RELMβ^−/−^ mice have decreased trans-epithelial electrical resistance (TEER) and increased permeability to (4-kDa) dextran in whole intestinal mounts (36). RELMβ stimulation of rat jejunal tissue promoted glucose transport mediated by alteration of glucose transporter proteins (diminished SGLT-1 and increased GLUT2 expression) and activation of PKC and AMPK signaling (184). Thus, the function of RELMβ in colitis is, at least in part, due to its ability to induce expression of RegIII proteins, modulate the intestinal microbiota, and influence epithelial permeability.

Other RELMs, including RELMα, usually restricted to immune cells (e.g., macrophages and dendritic cells) (169, 170), are expressed by epithelial cells, macrophages, and eosinophils during *C. rodentium* infection (185). However, RELMα could promote gut inflammation without microbicidal activities. RELMα^−/−^ mice infected with *C. rodentium* did not show higher pathogen burden, but decreased colitis with decreased CD4+ T cell expression of pro-inflammatory IL-17A (185). Additionally, intraperitoneal injection of mice with recombinant RELMα increased *C. rodentium-*induced colitis, including increased IL-17, whereas IL-17A^−/−^ mice did not display increased colitis in response to RELMα treatment (185). These data demonstrate a pro-inflammatory role of RELMα during *C. rodentium* infection, working mainly through the induction of IL-17. Similarly, RELMα^−/−^ mice have decreased inflammation during DSS colitis (186, 187). Thus, influence of RELMs on intestinal inflammation and infection is complex and involves direct antimicrobial activity, regulation of intestinal RegIII C-type lectins, modulation of the microbiota, and potential direct immunomodulatory effects.

## Conclusions

Aspects of major HDPs (i.e., cathelicidins, β-defensins, and members of RegIII and RELM families) in the colon and their relevance in pathogenesis of infectious colitis reviewed herein aid to uncover roles of these peptides in promoting epithelial defenses beyond direct microbial elimination (Figure 1). The presence of HDPs abundantly secreted into the intestinal lumen by epithelial cells and leukocytes during inflammation must be critical components of the innate immune response against enteropathogenic bacteria. They are bactericidal against enteric pathogens as well as the microbiota, while simultaneously modulating numerous cellular responses including leukocyte chemotaxis and migration, wound healing, cytokine production and pathogen sensing (Tables 1, 2). In fact, crude colonic mucus isolates from uninfected mice had no direct antibacterial activity against *C. rodentium* (96). Thus, there is increasing interest to decipher major mechanisms of HDPs in innate defense, which seem to be largely attributed to immunomodulatory functions.

To date, our understanding of HDP function in the colon is mostly limited to studies using mice genetically deficient in a single HDP, or via stimulation of mice with a specific exogenous peptide (commonly a synthetic peptide derived from an endogenous HDP). We propose a theoretical framework of how these peptides may work together in the gut. Host defense peptides would form an interactive peptide network capable of preventing colonization of enteropathogens by: (1) direct bacterial killing and (2) fine-tuning of the host immune response in the colon (i.e., modulation of epithelial cell responses and migration of leukocyte populations to the site of infection). In these activities, HPDs likely have overlapping and potentially complementary roles. Cathelicidins from different species displayed synergistic antibacterial activity against *P. aeruginosa, E. coli, E. faecalis*, and MRSA (188). Additionally, cathelicidins demonstrated synergistic antibacterial ability with human lysozyme (188). Thus, in the real scenario of numerous HDPs co-existing with other innate factors (e.g., MUC2 mucin, lysozyme), antibacterial activity against specific pathogenic species is likely enhanced.

The synergistic effects of multiple HDPs could apply to cytokine and chemokine signaling in the gut. Combined hBD-3 and LL-37 showed a synergistic reduction in secretion of proinflammatory factors (GRO-α, G-CSF, MCP-1, and IL-6) in gingival fibroblast-epithelial cells exposed to LPS, although they displayed only additive effects reducing IL-8 secretion (189). Other synergies can be predicted to occur during infectious colitis based on the effects of single HDPs. RELMβ regulates T-cell migration, IL-22 production and RegIIIγ in the colon (35–37), while cathelicidins, β-defensins, and RELMβ have all been demonstrated to regulate both mucus production and epithelial permeability (9, 11, 14, 15, 36, 38, 137, 138). This HDP network may decode how the intestinal innate immune system functions to quickly restore gut homeostasis and avoid damaging inflammation associated with pathogen colonization. Exploring how these peptides act synergistically in innate immune defenses and the complex signaling networks they activate during infectious colitis should lead to identification of therapeutic anti-infectious targets, or development of synthetic HDPs that work in combination to resolve intestinal infections. Such synthetic HDPs could be used either alone or in combination with reduced doses of antibiotics. These strategies of infectious disease control would be greatly beneficial, as emergence of antimicrobial resistance is rendering conventional antibiotics use unsustainable.

## Author Contributions

All authors listed have made a substantial, direct and intellectual contribution to the work, and approved it for publication.

## Conflict of Interest

The authors declare that the research was conducted in the absence of any commercial or financial relationships that could be construed as a potential conflict of interest.
